# Supplementary material for: The interactome of intact mitochondria by cross-linking mass spectrometry provides evidence for coexisting respiratory supercomplexes
Source: Mol Cell Proteomics. 2017 Dec 8;17(2):216–32. doi: 10.1074/mcp.RA117.000470 (PMC5795388; doi:10.1074/mcp.RA117.000470)
Supplement: Supplemental Data [file supp_RA117.000470_133922_0_supp_23978_fzffwf.zip › spectra_annotation/mito_spectra_annotation/239-1-3-1-5-1.pdf]

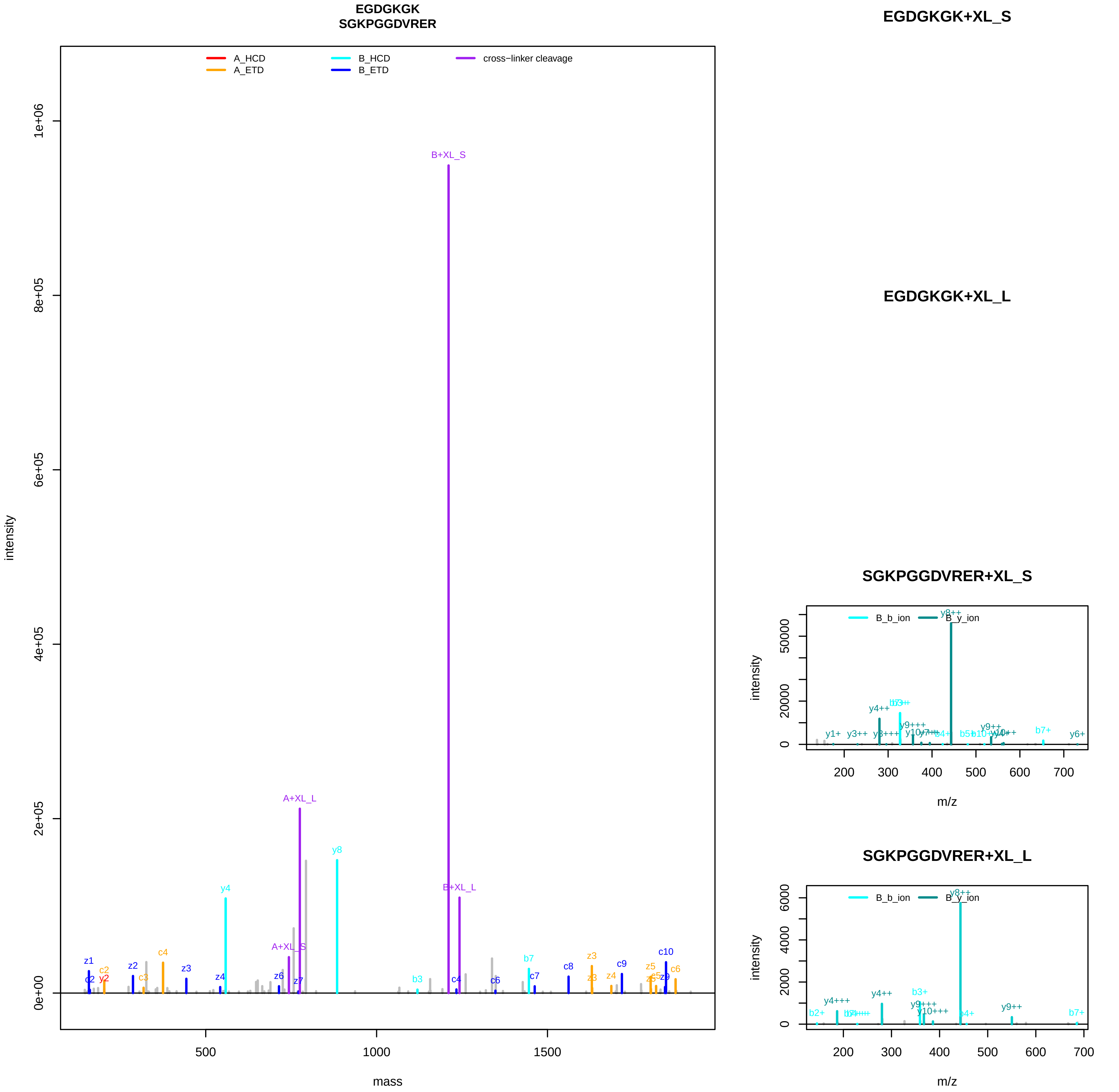

EGDGKGK
SGKPGGDVRER
A_HCD
A_ETD
B_HCD
B_ETD
cross−linker cleavage
B+XL_S
A+XL_L
B+XL_L
A+XL_S
y8
y4
b3
b7
c4
c6
c7
c8
c9
c10
z1
z2
z3
z4
z6
z7
c2
y2
c3
z5
c5
intensity
mass
EGDGKGK+XL_S
EGDGKGK+XL_L
SGKPGGDVRER+XL_S
SGKPGGDVRER+XL_L
B_b_ion
B_y_ion
y8++
y4++
b3++
y9++
y1+
y3++
b7+
y6+
y4+++
y10+++
b2+
b4+
y9+++
m/z
